# Detection of non‐criteria autoantibodies in women without apparent causes for pregnancy loss

**DOI:** 10.1002/jcla.22994

**Published:** 2019-08-11

**Authors:** Hongyuan Zhu, Meng Wang, Ying Dong, Honghai Hu, Qiaoli Zhang, Chen Qiao, Xin Xie, Fei Fan, Jiazi Zeng, Yan Jia, Lu Chen, Jingrui Liu, Lin Li, Yanhong Zhai, Zhen Zhao, Min Shen, Zheng Cao

**Affiliations:** ^1^ Department of Laboratory Medicine, Beijing Obstetrics and Gynecology Hospital Capital Medical University Beijing China; ^2^ Guangzhou Kangrun Biotech Co. Ltd. Guangdong China; ^3^ Department of Human Reproductive Medicine, Beijing Obstetrics and Gynecology Hospital Capital Medical University Beijing China; ^4^ Central Laboratory, Beijing Obstetrics and Gynecology Hospital Capital Medical University Beijing China; ^5^ Department of Pathology and Laboratory Medicine Weill Cornell Medicine New York NY USA; ^6^ Reference Laboratory MedicalSystem Biotechnology Co., Ltd. Ningbo China

**Keywords:** antiphospholipid, APS, autoantibody, miscarriage, pregnancy loss

## Abstract

**Background:**

Some of the non‐criteria autoantibodies, especially non‐conventional antiphospholipid (aPL) antibodies, were present with high prevalence in sporadic miscarriages and recurrent pregnant loss. However, whether these autoantibodies are associated with miscarriage patients without apparent causes remain unclear.

**Methods:**

The subjects were recruited from the female patients visiting the Infertility Center at the Beijing Obstetrics and Gynecology Hospital from January 2017 to March 2018. The women who experienced one sporadic miscarriage (n = 89) or recurrent pregnancy loss (n = 125) were enrolled. The control participants (n = 59) were those women with normal pregnancy history and with no miscarriage or thrombosis experience. The collected serum specimens from above patients and controls were subjected to the 13 non‐criteria autoantibody examinations, targeting non‐conventional phospholipids, thyroid, sperm, endometrial, and anti‐nuclear antigens.

**Results:**

When compared with the controls, the following non‐criteria antibodies stood out in present study with significantly increased frequency and were listed in the order of decreasing positive rates: aPE IgM (40.0%), ANA (15.2%), aEM IgG (13.6%), aPE IgG (12.8%), and aPT IgM (10.4%). Except for ANA, the presence of aPE IgM, aEM IgG, aPE IgG, and aPT IgM was not associated with positivity of LA tests. In receiver operating characteristic analyses, the combined aPE IgG and aEM IgG biomarker panel had the best discriminating power between miscarriage patients and healthy controls.

**Conclusion:**

Our findings suggested that the non‐criteria could be included as part of the pregnancy loss evaluation when apparent causes are absent, and the conventional aPLs tests failed to provide interpretations.

**Abbreviations**
aAnxV: anti‐annexin V
aCL: anti‐cardiolipin
aEM: anti‐endometrium
ANA: anti‐nuclear antibodies
aPC: anti‐phosphotidylcholine
aPE: anti‐phosphotidylethanolamine
aPI: anti‐phosphotidylinositol
aPL: antiphospholipid
APS: antiphospholipid syndrome
aPS: anti‐phosphotidylserine
aPT: anti‐prothrombin
aPTT: activated partial thromboplastin time
ASA: anti‐sperm antibody
ASRM: American Society for Reproductive Medicine
aTG: anti‐thyroglobulin
aTPO: anti‐thyroid peroxidase
AUC: area under curve
aβ2‐GPI: anti‐β2 glycoprotein I
DRVVT: dilute Russell's viper venom time
HLA: human leukocyte antigen
IFA: indirect immunofluorescence assay
LA: lupus anticoagulant
OR: odds ratio
ROC: receiver operating characteristic
RPL: recurrent pregnancy loss
SLE: systemic lupus erythematosus

## 1 INTRODUCTION

Spontaneous pregnancy loss is common, and it happens in 15%‐25% of all clinically recognized pregnancies.^1^, ^2^ Sporadic losses mostly result from chromosomal errors such as trisomy, monosomy, and polyploidy.^2^ Recurrent pregnancy loss (RPL), in contrast, is considered as a distinct clinical entity. Historically, RPL was defined as a disorder of three or more consecutive pregnancy failures prior to 20th gestational week, and it only affected 1% of couples aiming to conceive.^1^ Later on, according to the criteria set by the American Society for Reproductive Medicine (ASRM),^2^ RPL was re‐defined as two or more consecutive miscarriages excluding molar, ectopic, and biochemical pregnancies. Approximately 5% of women are estimated to suffer RPL.^3^, ^4^ The commonly accepted RPL risk factors include cytogenetic abnormalities, endocrine abnormalities, anatomical factors, immunological factors, inherited thrombophilia, infectious disease, malefactors, and miscellaneous factors such as psychological, lifestyle, environmental, and occupational factors.^1^, ^2^, ^3^, ^4^, ^5^

Several lines of evidence have shown that the presence of autoimmune diseases as well as their associated autoantibodies increased the risk of pregnancy loss.^6^, ^7^ The most well‐known autoimmune condition is the antiphospholipid syndrome (APS) which has been proven to be associated with RPL. The laboratory diagnosis of APS requires at least one of the three following conventional antiphospholipid antibody (aPL) assays tested positive: lupus anticoagulant (LA), anti‐cardiolipin (aCL) antibody, and anti‐β2 glycoprotein I (aβ2‐GPI).^8^ These three aPLs are by far the most widely accepted tests for APS diagnosis.^2^ Recently, more and more studies focused on the role of non‐conventional aPLs in RPL patients. For instance, anti‐phosphotidylethanolamine (aPE), anti‐phosphotidylserine (aPS), anti‐phosphotidylinositol (aPI), anti‐phosphotidylcholine (aPC), anti‐prothrombin (aPT), and anti‐annexin V (aAnxV) antibodies have been reported to be frequently associated with recurrent miscarriage and infertility.^7^, ^9^, ^10^, ^11^, ^12^ Besides non‐conventional aPLs, other non‐criteria autoantibodies were shown to be tied with RPL in various studies. According to the study by Ohmura et al,^13^ the prevalence and titer of anti‐C1q were significantly higher in unexplained RPL patients suggesting excessive complement activation process. Anti‐phosphatidylserine‐dependent/anti‐prothrombin (aPS/PT) antibody was shown to not only have higher prevalence in RPL patients but also be associated with adverse obstetric outcomes.^14^ It has been documented by several studies that thyroid autoimmunity was associated with RPL and infertility by disturbing the regular maturation of oocytes and fetal development.^12^, ^15^, ^16^ Anti‐nuclear antibodies (ANA), which is a routine screening test for systemic lupus erythematosus (SLE), were found to be increased in RPL patients according to a review published in 1996.^17^ Similarly, anti‐sperm antibody (ASA)^7^, ^18^ and anti‐endometrium (aEM) antibody^7^ resulting in implantation failure and endometriosis, respectively, have also been shown to be attributable to infertility. Even with all the endeavors to identify autoantibodies associated with miscarriage, most previous studies did not generate sufficient or consistent results except for conventional aPLs (LA, aCL, and aβ2‐GPI).

As a continuing effort of relating autoimmunity with pregnancy, 13 different immunoassays were performed for non‐criteria autoantibody detections in women with sporadic miscarriage or RPL, including non‐conventional aPLs, thyroid autoantibodies, ASA, and anti‐endometrial autoantibodies. More specifically, aPT IgG, aPT IgM, aAnxV IgG, aAnxV IgM, aPS IgG, aPS IgM, aPE IgG, aPE IgM, anti‐thyroglobulin (aTG) IgG, anti‐thyroid peroxidase (aTPO) IgG, anti‐sperm IgG, aEM IgG, and ANA were tested in present study. The odds ratio (OR) and other proper statistical parameters were calculated and compared between the miscarriage and the control groups.

## 2 MATERIALS AND METHODS

### 2.1 Patients

The subjects were from the female patients visiting the Infertility Center at the Beijing Obstetrics and Gynecology Hospital from January 2017 to March 2018. As part of the standard care by the Infertility Center, the patients who experienced one sporadic clinical pregnancy loss or RPL (two or more consecutive pregnancy loss) were all screened by the aCL and aβ2‐GPI ELISA assays. Only the subjects with negative screening results were recruited, in combination with the following exclusion criteria: definitive APS and autoimmune diseases, ectopic pregnancy, elective abortion, uterine abnormalities, chromosomal abnormalities, thyroid dysfunction, endocrine abnormalities, and genital tract infection. A clinical pregnancy is defined as an intrauterine pregnancy confirmed by ultrasound or histology evidence.^19^ Remaining serum specimens from the aCL and aβ2‐GPI screening experiments were stored and examined for the non‐criteria autoantibodies described in present work. With the patients' consents, the LA tests were performed for totally 70 enrolled patients (30 patients with one sporadic miscarriage and 40 with RPL) with their citrated plasma specimens. As there is no specific term for women who experienced non‐consecutive pregnancy losses interspersed with normal pregnancies, only the patients with one sporadic miscarriage or with RPL were enrolled. The control participants were those women with normal pregnancy history and with no miscarriage or thrombosis experience. The controls were recruited from the outpatients visiting our hospital for pre‐pregnancy evaluation.

This study was approved by the Ethics Committee of Beijing Obstetrics and Gynecology Hospital (approval number: 2016‐KY‐075‐01). Two‐milliliter serum was collected from each of the recruited patients and controls, and 2 mL citrated plasma was collected at the same time for the 70 patients with their consents.

### 2.2 Reagents and methods

The following commercial ELISA kits were used for detection of autoimmune antibodies, including aCL IgA/G/M (AESKU Diagnostics, Germany, Ref 3202), aβ2‐GPI IgA/G/M (AESKU Diagnostics; Ref 3215), aPE IgG and IgM (AESKU Diagnostics; Ref 3209), aPS IgG and IgM (AESKU Diagnostics; Ref 3207), aPT IgG and IgM (AESKU Diagnostics; Ref 3229), aAnxV IgG and IgM (AESKU Diagnostics; Ref 3240), aTG IgG (AESKU Diagnostics; Ref 3400), aTPO IgG antibodies (AESKU Diagnostics; Ref 3401), anti‐sperm IgG (Anqunshengwu; Ref 0.0.0056), and aEM IgG (Anqunshengwu; Ref 0.0.0060). All ELISA assays were performed in the institutional clinical laboratory, and the experimental steps were briefly described as follows. The diluted sera were incubated in 96‐well microplates enclosed in the ELISA kits for 30 minutes at room temperature. After the washing step, the conjugate was incubated and washed again before adding the substrate to generate enzymatic colorimetric reactions. The concentration of target antibody was calculated based on its OD (at the wavelength of 450 nm) value compared with the standard curve. The lupus anticoagulant tests (dilute Russell's viper venom time, DRVVT) were performed on the Werfen ACL TOP 500 coagulation analyzer with a screening/confirmation ratio cutoff value of 1.20 provided by the manufacturer (Instrument Laboratory; Ref 0020301500/0020301600).

The ANA reactivity was determined with the indirect immunofluorescence assays (IFAs; AESKU Diagnostics; Ref 51.100) at the starting dilution fold of 1:80 for collected serum samples. All ANA IFAs were performed on the automatic IFA system HELIOS (AESKU Diagnostics) according to the manufacturer's instructions. Briefly, the diluted sera were incubated on the Hep‐2 cell‐coated slides for 30 minutes at room temperature. After washing off the non‐specific binding, the FITC‐conjugated anti‐human IgG was added for another incubating step followed by the mounting medium application. The fluorescent images were captured and analyzed by the HELIOS software.

### 2.3 Statistical analysis

Statistical analyses were performed with the SPSS 22.0 (IBM) software. Statistical significance of results was assessed using the student's *t* test, the chi‐square test, or the odds ratio (OR) with SPSS. The *P* values <.05 were considered to have statistical significance. The receiver operating characteristic (ROC) curves were performed with the SigmaPlot (Systat Software Inc) software.

## 3 RESULTS

### 3.1 Patient recruitment

**Figure 1:**
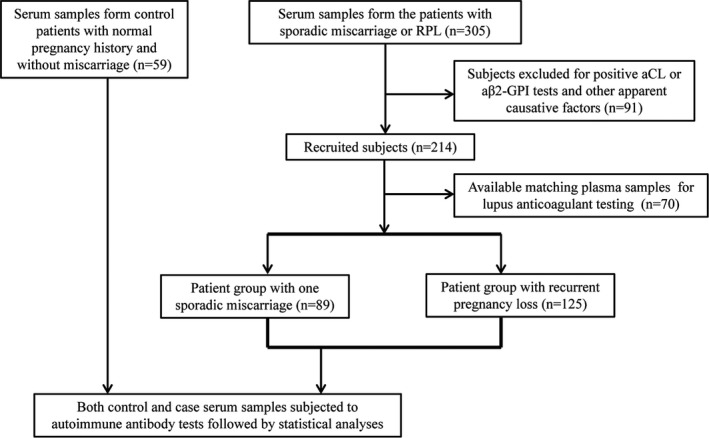
Schematic diagram for patient recruitment and study design

The patient recruitment criteria and the clinical laboratory study workup flow were schematically shown in Figure 1. After excluding the subjects who had obvious clinical or genetic conditions that are considered high‐risk factors for pregnancy loss, totally 214 out of 305 patients were eventually enrolled in present study as the positive cases. Of the recruited case subjects (n = 214), eighty‐nine patients had one previous sporadic miscarriage, and 125 patients had at least two consecutive pregnancy loss (or RPL). Of the RPL patients, 97 experienced two miscarriages, and 28 had three or more miscarriages. No matter with sporadic or RPL, the majority of the miscarriage took place in the first trimester (Table 1). The negative control subjects (n = 59) were the patients who had normal pregnancy histories and experienced no pregnancy loss or thrombosis before. As summarized in Table 1, compared with the control group, younger mean ages of the patients with one sporadic miscarriage and two pregnancy loss were observed (*P* < .05, student's *t* test).

**Table 1:** Demographic table for the controls and the patients with miscarriages

	Control	One sporadic miscarriage	Recurrent pregnancy loss	
			Two PL	Three or more PL
Number	59	89	97	28
Age (mean ± SD)	32.5 ± 3.5	31.0 ± 4.1	30.1 ± 4.3	32.3 ± 4.5
Trimester of last PL				
First trimester	–	84	94	26
Second trimester	–	5	3	2

Abbreviation: PL, pregnancy loss.

### 3.2 Laboratory testing and statistical analyses

As shown in Figure 1, the case patients' sera (n = 305) were all initially applied to the conventional aCL and aβ2‐GPI antibody tests, which are recognized as two of the screening experiments for APS patients. Only the subjects that had negative aCL and aβ2‐GPI screening results and met the other described excluding criteria were enrolled (n = 214) for the next‐step immunoassays targeting autoantibodies against non‐conventional phospholipids, thyroid, sperm, endometrium, and nuclear antigens.

The positive rates of the listed autoantibody experiments were summarized in Table 2, with the cutoffs provided by the manufacturers (Table 2). Among these tests, the aPT IgM, aPE IgG, aPE IgM, aEM IgG, and ANA presented positive rates >10% in the patient group with one sporadic miscarriage and/or in the RPL group. Importantly, according to the chi‐square analysis results (Table 3), all the above five antibodies showed significantly higher prevalence (*P* < .05) in sporadic miscarriage or RPL patients when compared with healthy controls. By contrast, 10.4% of the RPL patients were tested aTPO IgG positive (Table 2) but there was lack of significant difference in prevalence between groups (data not shown).

**Table 2:** Positive rates of the autoantibodies in different patient groups

Autoimmune antibodies	Cutoff	Control (n = 59)		One sporadic miscarriage (n = 89)		Recurrent pregnancy loss (n = 125)	
		Positive rate	Mean ± SD	Positive rate	Mean ± SD	Positive rate	Mean ± SD
aPT IgG	18 U/mL	1.6% (1)	2.8 ± 3.5	3.4% (3)	5.1 ± 6.9	1.6% (2)	4.5 ± 4.1
aPT IgM	18 U/mL	0.0% (0)	3.8 ± 2.9	9.0% (8)	8.1 ± 7.6	10.4% (13)	9.0 ± 7.9
aAnxV IgG	18 U/mL	0.0% (0)	1.1 ± 1.2	2.3% (2)	4.2 ± 5.9	0.8% (1)	3.1 ± 2.4
aAnxV IgM	18 U/mL	1.7% (1)	1.4 ± 6.7	1.1% (1)	2.4 ± 6.4	1.6% (2)	2.2 ± 4.2
aPS IgG	18 U/mL	0.0% (0)	1.8 ± 1.0	2.3% (2)	5.9 ± 7.3	1.6% (2)	5.1 ± 9.1
aPS IgM	18 U/mL	0.0% (0)	1.2 ± 0.6	4.5% (4)	6.3 ± 11.1	2.4% (3)	4.2 ± 4.6
aPE IgG	18 U/mL	0.0% (0)	5.0 ± 2.0	19.1% (17)	13.1 ± 9.4	12.8% (16)	18.3 ± 55.7
aPE IgM	18 U/mL	3.4% (2)	6.4 ± 4.0	23.6% (21)	12.8 ± 9.8	40.0% (50)	18.6 ± 14.9
aTG IgG	180 IU/mL	0.0% (0)	12.2 ± 16.9	5.6% (5)	39.5 ± 131.3	4.8% (6)	28.2 ± 106.4
aTPO IgG	60 IU/mL	5.1% (3)	11.2 ± 37.1	7.9% (7)	39.9 ± 136.5	10.4% (13)	52.5 ± 213.6
anti‐sperm IgG	S/Co = 1	3.4% (2)	0.2 ± 0.2	5.6% (5)	0.5 ± 0.9	5.6% (7)	0.4 ± 0.9
aEM IgG	S/Co = 1	1.7% (1)	0.3 ± 0.2	14.6% (13)	0.8 ± 0.4	13.6% (17)	0.7 ± 0.5
ANA	1:80 titer	0.0% (0)	ND	7.9% (7)	ND	15.2% (19)	ND

The positive rate of each autoimmune antibody tested was calculated as the percentage of the frequency of the positive results (indicated in parenthesis) over the total patient number of each group.

All cutoff values were provided by the manufacturers' package inserts.

Abbreviations: aAnxV, anti‐annexin V; aEM, anti‐endometrium; ANA, anti‐nuclear antibodies; aPE, anti‐phosphotidylethanolamine; aPS, anti‐phosphotidylserine; aPT, anti‐prothrombin; aTG, anti‐thyroglobulin; aTPO, anti‐thyroid peroxidase; ND, not determined; S/CO, signal‐to‐cutoff ratio.

**Table 3:** Statistical comparison of autoantibodies by chi‐square test and odds ratio

	Sporadic miscarriage vs control		RPL vs control		RPL vs sporadic miscarriage	
	Chi‐square *P*	Odds ratio (95% CI)	Chi‐square *P*	Odds ratio (95% CI)	Chi‐square *P*	Odds ratio (95% CI)
aPT IgM	.018	NA	.010	NA	.732	ND
aPE IgG	<.001	NA	.004	NA	.208	ND
aPE IgM	.001	8.8 (2.0‐39.2)	<.001	19 (4.4‐81.4)	.012	2.2 (1.2‐4.0)
aEM IgG	.009	9.9 (1.3‐78.0)	.011	9.1 (1.2‐78.0)	.834	ND
ANA	.027	NA	.003	NA	.105	ND

Only the autoimmune antibodies with statistically significant (*P* < .05) differences among patient groups were listed. NA: no available odds ratio can be calculated due to the presence of a zero value (meaning no positive results for a particular autoimmune antibody). ND: odds ratio was not determined when the corresponding chi‐square *P* value was >.05.

Abbreviations: aEM, anti‐endometrium; ANA, anti‐nuclear antibodies; aPE, anti‐phosphotidylethanolamine; aPT, anti‐prothrombin; CI, confidence intervals; RPL, recurrent pregnancy loss.

In addition, when patients were tested positive for aPE IgM or aEM IgG, they were more likely to experience sporadic miscarriage or RPL with their corresponding odds ratios >1.0 (ranged from 8.8 to 19.0) in Table 3. If compared with the group of one sporadic miscarriage, the RPL group displayed a higher prevalence only in the aPE IgM test with a chi‐square *P* value of .012 and an odds ratio of 2.2.

With patients' consents, we were able to perform the LA tests in our laboratory with their citrated plasma collected at the same time as the serum specimens of the available case subjects (n = 70). As shown in Table 4, except for ANA (*P* = .038), none of the listed antibodies exhibited significant difference in association with the positivity of LA testing.

**Table 4:** Statistical comparison of the autoantibodies in the lupus anticoagulant testing groups

Autoimmune antibodies	Lupus anticoagulant testing		Chi‐square *P*
	Negative (n = 63)	Positive (n = 7)	
aPT IgM	6.3% (4)	0.0% (0)	1.000
aPE IgG	17.5% (11)	28.6% (2)	.473
aPE IgM	38.1% (24)	14.3% (1)	.212
aEM IgG	19.0% (12)	14.3% (1)	.759
ANA	12.7% (8)	42.9% (3)	.038

The positive rate of each autoimmune antibody listed (aPT IgM, aPE IgG and IgM, aEM IgG, ANA) was calculated as the percentage of the frequency of the positive results (indicated in parenthesis) over the total patient number of each group that was based on the lupus anticoagulant testing results.

Abbreviations: aEM, anti‐endometrium; ANA, anti‐nuclear antibodies; aPE, anti‐phosphotidylethanolamine; aPT, anti‐prothrombin.

### 3.3 ROC curve analyses

**Figure 2:**
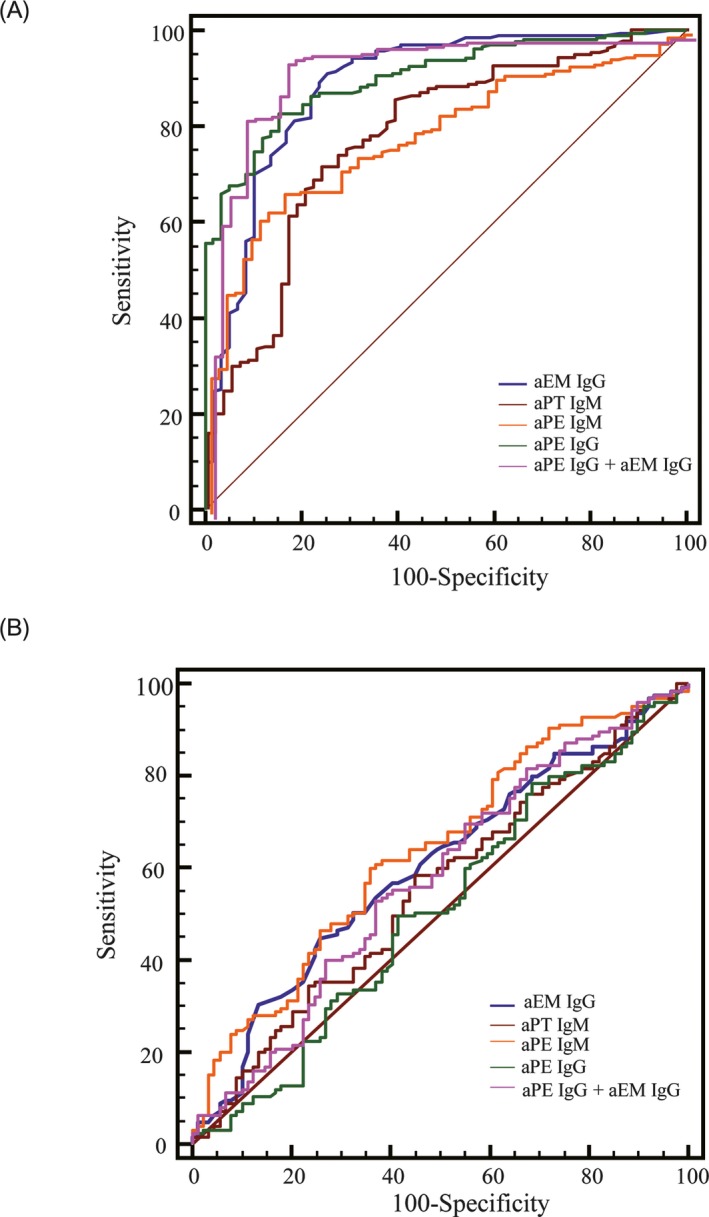
Receiver operating characteristic analyses of autoantibodies in differentiating patients with or without pregnancy loss (A) and differentiating patients with one sporadic miscarriage or recurrent pregnancy loss (B)

To examine their diagnostic power in differentiating patients with or without miscarriage(s) (one or more pregnancy loss), the ROC curve analyses (Figure 2A) were carried out for the aPT IgM, aPE IgG, aPE IgM, and aEM IgG tests with the corresponding area under curves (AUCs) calculated as follows: 0.780, 0.902, 0.795, and 0.886, respectively. The top two AUCs of aPE IgG and aEM IgG were statistically higher than those of aPT IgM and aPE IgM (*P* < .05, calculated with SPSS software). Interestingly, when the aPE IgG and aEM IgG tests were combined, the corresponding AUC (AUC = 0.947) is significantly higher than that of the individual aPE IgG or aEM IgG (*P* < .01). By contrast, however, in the ROC analyses for differentiating patients with sporadic miscarriage and RPL (Figure 2B), the corresponding AUCs for the aPT IgM, aPE IgG, aPE IgM, aEM IgG and combined aPE IgG and aEM IgG were 0.552, 0.508, 0.636, 0.600, and 0.575, respectively, indicating much lower efficiency in discriminating the above two groups. As shown in Table 5, with the maximized Youden's index, the cutoff values, sensitivity, and specificity were calculated for the above autoantibodies analyzed in ROC curves (Figure 2).

**Table 5:** The cutoff values, sensitivity, and specificity for the antibodies analyzed in the receiver operating characteristic curves

	Cutoff	Sensitivity (%)	Specificity (%)
aEM IgG	4 (S/Co)	1	100
aPT IgM	9 U/mL	34	76
aPE IgM	13 U/mL	61	63
aPE IgG	13 U/mL	33	71

The cutoff values were chosen when the Youden's index (sensitivity + specificity − 1) reached its maximum value.

## 4 DISCUSSION

Although great amount of efforts has been put into the etiology studies, it is reported that as many as 50%‐75% of RPL women present to the clinic without any apparent causative factor.^3^, ^4^ In present work, we found that aPT IgM, aPE IgG, aPE IgM, aEM IgG, and ANA antibodies were significantly associated with sporadic miscarriage and RPL patients without apparent causes. The combined aPE IgG and aEM IgG biomarker panel had the best discriminating power between miscarriage patients and healthy controls.

### 4.1 Sporadic miscarriage and RPL

Both sporadic miscarriage and RPL are frustrating and they together affect a significant portion of pregnant women.^19^ According to the ASRM's recommendation, RPL was defined as two or more consecutive pregnancy losses and should be recognized as a distinct disorder when compared with sporadic miscarriage.^2^ Although maternal age is a well‐known miscarriage risk factor due to increased incidence of chromosomal anomalies, the frequency of aneuploidy in RPL patients was lower than in those with sporadic miscarriages^20^, ^21^ at the same age. Similar observation was made with fetal aneuploidy which was less common in RPL cases than in sporadic miscarriage controls.^22^ Aside from the genetic causes, various risk factors have been identified and linked with both sporadic miscarriages and RPL, such as endocrine dysfunctions, infections, immunology disorders, and so on.^2^, ^3^, ^19^, ^23^

### 4.2 Autoantibodies in sporadic miscarriage and RPL

In risk factor studies for miscarriage, autoimmune disorders or dysfunctions were widely accepted as one of the suspected causes although there has been no solid proof that they have harmful effect on pregnancy.^2^, ^19^ The APS, which is one of the most well‐studied autoimmune disorders,^1^, ^2^, ^3^, ^6^, ^23^ is associated with RPL and has been recommended to be included in RPL evaluation and treatment by the ASRM in 2012.^2^ Besides the clinical presentations in thrombosis or pregnancy loss, the laboratory APS diagnosis requires two positive detections 12 weeks apart of the three conventional aPLs: aCL, aβ2‐GPI, and LA. In pregnancy, aPLs interfere with trophoblast syncytium formation which may have direct effects on placental structures and promote placental thrombosis and fetal loss.^24^ It has been shown that the aβ2‐GPI directly binds to the cultured cytotrophoblast cells in vitro, triggering an inflammatory response that led to trophoblast damage.^2^, ^25^

In addition to the above conventional aPLs, a spectrum of so‐called non‐conventional aPLs, such as aPT, aAnxV, aPS, aPE, were shown to be associated with APS caused pregnancy loss.^2^, ^9^, ^26^, ^27^ However, due to lack of standardization between testing laboratories and insufficient clinical evidence, it has been controversial whether or not the non‐conventional aPLs should be listed as independent testing criteria. Neither the ASRM RPL practice guideline^2^ nor the international APS diagnosis consensus^8^ recommends routine screening for the non‐conventional aPLs. However, the non‐criteria autoantibody tests were recommended for RPL women with relevant clinical manifestations according to the guideline published by the German Society of Gynecology and Obstetrics.^23^

In present study, the women of sporadic miscarriage or RPL without apparent causes were recruited. All the enrolled subjects (n = 214) were tested negative in the conventional aPL screening assays for aCL and aβ2‐GPI. Then, we further examined the patients' autoimmune status with 13 autoantibodies targeting phospholipids, thyroid, sperm, endometrium, and nuclear antigens. When compared with controls, the following antibodies stood out with significantly increased frequency and were listed in the order of decreasing positive rates in RPL group: aPE IgM (40.0%), ANA (15.2%), aEM IgG (13.6%), aPE IgG (12.8%), and aPT IgM (10.4%; Table 2). The term “seronegative APS” or “non‐conventional APS” was previously described for patients with obstetrical and/or thrombotic manifestations but with negative detection of LA, aCL, or aβ2‐GPI. In the study by Mekinian et al^9^ in which they tested non‐conventional aPLs in seronegative APS and confirmed APS groups, aAnxV IgG, aPE IgG, aPE IgM, aPS/PT IgG, and aPS/PT IgM were all identified in both groups. However, the aPS/PT IgG and aPS/PT IgM were much more significantly elevated in the group of confirmed APS than in the seronegative APS patients. In their ROC analysis, the aAnxV IgG and the aPE IgG were the best biomarker discriminating the seronegative patients from healthy controls, with AUC >0.8,^9^ suggesting the usefulness of the non‐conventional aPLs when evaluating suspected APS patients in the absence of LA, aCL, or aβ2‐GPI. According to Mekinian's definition, some of our recruited subjects may be categorized as seronegative APS patients. However, due to lack of assay standardization in reagents' specificity and cutoff values, it would be unlikely to make direct comparison for the performance of non‐conventional aPLs examined in Mekinian's and our experiments.

The aAnxV antibodies recognize the free form of the potent anticoagulant aAnxV and were found to be a risk factor for early pregnancy loss.^9^, ^11^, ^27^ With our patients, the prevalence of aAnxV IgG and IgM was low in both of the case and healthy control groups. In other studies, the aAnxV antibodies were found to be elevated in both RPL patients and controls with similar frequencies^28^ and could not be used as a risk factor for RPL.^29^ The above discrepant findings of the aAnxV antibodies truly represent a general barrier for non‐criteria aPLs application in miscarriage studies. The aPT antibodies were well‐documented to be associated with adverse pregnancy outcomes such as pregnancy loss, although aPT IgG seemed more sensitive than IgM in those studies.^30^, ^31^ The mechanism by which aPT antibodies could increase miscarriage risk has not been well understood. It was proposed that aPT antibodies might promote microvascular placental thrombosis by cross‐linking prothrombin on the cell surface and interfering the downstream signaling pathways.^30^ The aPS antibodies, with similar observation in present study, were not reported to be associated with recurrent miscarriage and unexplained fetal losses.^32^ Interestingly, the aPS/PT autoantibodies targeting the phosphatidylserine/prothrombin complex were shown to have higher positive rates in the APS patients with pregnancy morbidity^9^, ^30^ and therefore were more widely studied in the field of reproduction medicine. As another stronger risk factor for early and mid‐to‐late pregnancy loss, the aPE antibodies have been shown to be significantly increased in women with RPL.^11^, ^26^, ^33^ In our study, aPE antibodies (IgG and IgM) were vastly elevated in both sporadic miscarriage and RPL patients (Table 2) compared with the control group. Notably, the aPE IgM is more closely associated with RPL than with sporadic miscarriage (*P* = .012, odds ratio 2.2). It was reported that aPE antibodies directly recognized PE‐binding proteins such as kininogen and resulted in thrombosis by thrombin‐induced platelet aggregation.^26^

Endometriosis is the abnormal presence of endometrial tissue in ovaries, and other ectopic locations, with pelvic pain and infertility as its major symptoms.^7^ The aEM IgG targeting laminin‐1 was found to interfere with embryo early pre‐implantation and organogenesis after implantation.^7^ More specifically, the aEM IgG was reported to be significantly associated with recurrent early miscarriages and subsequent adverse pregnancy outcomes.^34^ Our results also showed that aEM IgG was a risk factor for both sporadic miscarriage and RPL patients (Table 3). The AUC of aEM IgG was 0.886 in the ROC analysis, indicating a good discriminating power between patients with miscarriages and healthy controls (Figure 2A). In contrast, the anti‐sperm antibodies were not associated with pregnancy loss according to an earlier prospective study^35^ and its presence mainly resulted in reproductive failure.^7^

The presence of thyroid autoantibodies is associated with clinical hyperthyroidism and hypothyroidism. Several studies further supported that thyroid autoimmunity increased the risk of complications of pregnancy loss, recurrent miscarriage, and preterm delivery.^6^, ^15^, ^36^ It was reported that the thyroid antibodies were found in 5%‐15% of women at reproductive age without thyroid dysfunctions.^6^ We observed moderately increased aTPO IgG in all three groups including healthy controls but found no significant difference between those groups. The ANAs are a group of autoantibodies targeting nuclear antigens in human cell and have been detected in a series of autoimmune disorders. More importantly, the ANA has been reported to be highly elevated in both unexplained and explained RPL patients with the positive rates ranged 34%‐51%.^37^, ^38^, ^39^ With our patients, The ANA was the only non‐phospholipid antibody that was significantly elevated compared with the controls, with a higher prevalence in the RPL group (15.2%) than in the sporadic miscarriage group (7.9%; Table 2).

The LA test is one of the three recommended laboratory tests for APS diagnosis. And there was evidence supporting that LA correlates with thrombosis and pregnancy morbidity.^8^ However, unlike aβ2‐GPI and aCL recognizing specific antigens which are plasma protein β2‐GPI and the phospholipid cardiolipin, respectively, LA can be caused by a group of antiphospholipid antibodies directly against negatively charged phospholipids or complexes between phospholipids and proteins. Even after decades of appearance, the identity of the responsible antigens still remains unsolved.^40^

With the consideration of the citrated plasma availability, we were able to perform the LA testing on 70 case patients, of which 30 were from the sporadic miscarriage group and 40 were from the RPL group. As seen in Table 4, only 10% (n = 7) of the tested patients were LA positive, the frequency of which was in line with our historical LA results for patients with miscarriages. We need to be aware that according to the APS diagnosis consensus, the LA positive results need to be repeated in two separate occasions 12 months apart.^8^ In the present context, the phrase “LA positive” indicates only one‐time positive results observed in our study.

According to the recent International Society on Hemostasis and Thrombosis (ISTH) and Clinical and Laboratory Standards Institute (CLSI) guidelines for the LA laboratory detection, both DRVVT and activated partial thromboplastin time (aPTT) should be tested and either positive result may lead to LA detection.^41^ Therefore, DRVVT and aPTT might be both used as exclusion criteria to confirm the absence of lupus anticoagulant or even APS in our patients. However, aPTT is not included in our routine LA testing panel but was commonly ordered for miscarriage patients' coagulation evaluation. Of the 63 DRVVT‐negative patients, only two had elevated aPTT and they could be LA positive if confirmatory assay such as platelet neutralization procedure confirms it. In our study, except for ANA, the presence of the rest antibodies in Table 4 did not seem to be associated with the positive detection of LA. The ANA was more likely to be elevated in LA positive patients. Interestingly, in a study conducted with primary APS patients, the ANA was found to be positive in about 30% of APS patients who were tested LA positive.^42^ An earlier relevant report suggested that ANA positivity may pose a higher risk of deep vein thrombosis in the APS patients.^43^

A few limitations exist in present study. Due to limited access to citrated plasma of the recruited patients, not all subjects were tested with LA experiments. Although we have excluded the patients with confirmed APS using their medical records, there might be a small portion of APS patients mixed into the cohort. The exact number of APS patients recruited was not known but should be lower than 10% of total miscarriage patients (LA positive rates were 10% in present study). According to our study protocol, the patients with definitive autoimmune diseases (such as SLE) had been excluded. However, as this is a retrospective study performed with the outpatients at our Infertility Center and the complete laboratory evaluation was not performed for autoimmune diseases, a small number of patients with subclinical autoimmune disease(s) might be included and could be a confounding factor to the results.

Another limitation is that we could not determine the definitive cutoff values for the autoantibodies examined in present study, due to the sensitivity and specificity difference of ELISA reagents used across the laboratories focusing pregnancy loss. It was a common hurdle that prevents the non‐criteria autoantibodies screening from universal application for routine miscarriage evaluation. The Mekinian^9^ group proposed the cutoffs for non‐conventional aPLs in the study of differentiating APS and non‐APS patients. The authors also commented that clinical laboratories need to establish their own non‐traditional aPLs cutoffs based on the specific clinical subsets and the assay reagents.

## 5 CONCLUSION

In summary, our findings suggested that the non‐criteria antibodies could be included as part of the pregnancy loss evaluation when apparent causes are absent, and the conventional aPLs tests failed to provide interpretations.
